# Improved Purification of Human Granzyme A/B and Granulysin Using a Mammalian Expression System

**DOI:** 10.3389/fimmu.2022.830290

**Published:** 2022-03-01

**Authors:** Valerio Rasi, Owais Abdul Hameed, Patricia Matthey, Sibes Bera, Duane P. Grandgenett, Stefan Salentinig, Michael Walch, Daniel F. Hoft

**Affiliations:** ^1^ Department of Molecular Microbiology and Immunology, Saint Louis University School of Medicine, Saint Louis, MO, United States; ^2^ Department of Internal Medicine, Division of Infectious Diseases, Allergy and Immunology, Saint Louis University School of Medicine, Saint Louis, MO, United States; ^3^ Anatomy Unit, Department of Oncology, Microbiology and Immunology, Faculty of Science and Medicine, University of Fribourg, Fribourg, Switzerland; ^4^ Department of Chemistry, Faculty of Science and Medicine, University of Fribourg, Fribourg, Switzerland

**Keywords:** granzyme A, granzyme B, granulysin, cytotoxic granular proteins, mammalian expression, HEK293T, isotonic buffer, lipofectamine 3000

## Abstract

Cytotoxic lymphocytes release proteins contained within the cytoplasmic cytolytic granules after recognition of infected or tumor target cells. These cytotoxic granular proteins (namely granzymes, granulysin, and perforin) are key immunological mediators within human cellular immunity. The availability of highly purified cytotoxic proteins has been fundamental for understanding their function in immunity and mechanistic involvement in sepsis and autoimmunity. Methods for recovery of native cytotoxic proteins can be problematic leading to: 1) the co-purification of additional proteins, confounding interpretation of function, and 2) low yields of highly purified proteins. Recombinant protein expression of individual cytolytic components can overcome these challenges. The use of mammalian expression systems is preferred for optimal post-translational modifications and avoidance of endotoxin contamination. Some of these proteins have been proposed for host directed human therapies (e.g. - granzyme A), or treatment of systemic infections or tumors as in granulysin. We report here a novel expression system using HEK293T cells for cost-effective purification of high yields of human granzymes (granzyme A and granzyme B) and granulysin with enhanced biological activity than previous reports. The resulting proteins are free of native contaminants, fold correctly, and remain enzymatically active. Importantly, these improvements have also led to the first purification of biologically active recombinant human granulysin in high yields from a mammalian system. This method can be used as a template for purification of many other secreted cellular proteins and may lead to advances for human medicine.

## Introduction

The immune response to various intracellular pathogens and tumors includes cytotoxic T lymphocytes (CTLs) and natural killer (NK) cells which recognize and directly kill infected or malignant cells. They are involved in cell mediated immunity and play a major role in host defense against infections by intracellular pathogens including bacteria, viruses and fungi replicating in host cells (1). The effector molecules which kill host cells and intracellular microbial pathogens include a family of serine proteases (Granzymes or Gzms) and a small antimicrobial protein (Granulysin or GNLY) delivered by the pore-forming protein perforin (2).

There are five human granzymes (granzyme A, B, H, K, and M), and ten mouse counterparts. Based on substrate specificity studies, other groups have shown species specific immunological functions of granzymes highlighting a potential divergent evolution process and suggesting that the human counterparts ought to be used for translational work to human medicine (3). Thus, our work cited here will focus on human granzymes and GNLY. Furthermore, granzymes are ubiquitously expressed in CTL and NK cells, particularly GzmB which is even detected in non-cytotoxic immune cells (4, 5). As GNLY is not expressed in mice and therefore *in vivo* experiments to date have been performed only using human GNLY transgenic mice (6–8). Granzyme A (GzmA) was previously thought to only induce apoptosis of target cells in concert with perforin. Death induction by GzmA involves a complex sequence of events, ultimately leading to the activation and nuclear transfer of two nucleases (NM23-H1 and Trex1) that trigger lethal DNA damage (9–13). GzmA also induces monocytes to produce pro-inflammatory cytokines (14–16) and to inhibit the intracellular replication of mycobacterial growth within infected primary human monocytes (15, 17). Due to its proinflammatory potential, there have been recent reports of its involvement during bacterial sepsis (16, 18, 19). To further study these effects, it is imperative that researchers are careful to avoid potential endotoxin contamination in the final purified products: the use of a bacterial expression system will directly contaminate purified proteins, while any other system will contaminate the product if the researcher is not careful throughout the process. In contrast to human GzmA, Granzyme B (GzmB) induces apoptosis of the target cells either by direct or indirect activation of caspase 3 and 7 (20, 21). Furthermore, GzmB efficiently activates the mitochondrial death pathway by truncating the pro-apoptotic protein Bid (22) and induces mitochondrial outer membrane permeabilization (23). Ultimately, activated caspases trigger the release of an active DNase (CAD), responsible for DNA fragmentation and nuclear changes during apoptosis (24).

GNLY is a prokaryotic membrane-disrupting, lymphocytic effector protein (25). GNLY alone can kill a wide array of microbial pathogens when micromolar concentrations are used in cell culture (1). However, its intracellular antimicrobial activity is synergistically enhanced by GzmB (26, 27). Pathophysiological roles of these proteins have been reported demonstrating their broad implications for understanding immune homeostasis and pathogenic inflammatory diseases (28). Known functions of these lymphocytic effectors heavily relied on *in vitro* studies using purified proteins in killing assays as well as biochemical and morphological studies.

GzmA/B and GNLY were expressed and purified from various recombinant systems including bacteria (29–31), yeast (32) and insect cells (33). These systems produce proteins without the post-translational modifications identical to native cytotoxic effectors. Native purifications of granzymes (14, 34) and granulysin from NK cells and CTL cell lines like YT Indy and NK92MI yield limited amounts of purified proteins contaminated with other cytolytic granule components (35). The existing recombinant protein expression methods (e.g., bacteria, yeast, and insect) can yield higher concentrations and purity, but also alter tertiary structures and unnaturally absent glycosylations (36, 37). The mammalian expression system with HEK293 T cells was shown to produce recombinant GzmA, GzmB and GzmM proteins with higher molecular weights than their bacterial counterparts that can be cleaved using an endoglycosidase, suggesting that glycosylated proteins can be produced in a mammalian system (38).

We present an improved purification process for the purification of GzmA/B and GNLY that builds on previous protocols (35, 38). This was accomplished by modifying the transfection method, medium and buffer conditions, and enterokinase (EK) activation and purification steps. Our yields were consistently ten times higher with at least similar purity as compared to previous results (38). Our purified proteins are biologically active with higher specific activity as measured in diverse and complex immunological systems. We also highlight steps that minimize endotoxin contamination by using mammalian expression.

## Materials and Equipment

### 1 Production of the Expression Plasmid pHLsec-Gzm

- cDNA synthesis kit such as ThermoFisher Scientific RevertAid (#K1621).
- pHLsec plasmid (39).
- Gene synthesis companies (for GzmA studies, Genewiz was used).
  - Sanger sequencing for verification of inserted gene sequence within plasmids [use published primers that were in (38)]. Primer sequence for GNLY: Forward: pHLHisEKconsAge1For (5’-GAA-ACC GGT CAC CAC CAT CAC CAT CAC GAC GAC GAC GAC AAA) Reverse: pHLgnlySTOPkpn1Rev (5’-CTT GGT ACC TCA TTA CCT GAG GTC CTC ACA G) (Microsynth).
  - Standard gene synthesis using known nucleotide and protein sequence of target granzyme (using Uniprot and NCBI).
  - Optional: site-directed mutagenesis for modifications within original construct to obtain substitution at key amino acid [performed for GzmA-WT vs GzmA-S195A for (17)].

### 2 Expansion of HEK293T Cells

- 293T/17 (HEK293T/17) cells (ATCC #CRL-11268).
- 10 cm tissue culture dishes (Sigma # CLS430165) or T-75cm^2^ flasks (ThermoFisher Scientific #156499).
- Dulbecco’s Modified Eagle Medium (DMEM) (ThermoFisher Scientific #11965084).
- Stericup Quick Release (Sigma #S2GPU02RE).
- Fetal bovine serum (Sigma #F2442).
- Penicillin-Streptomycin (Gibco #15140-122).
- L-glutamine (Lonza #17-605E).
- Trypsin-EDTA (0.05%), phenol red (ThermoFisher Scientific #25300054).
- 2 M Trizma hydrochloride solution, pH 8 (Sigma #T3069-1L).
- 2 M CaCl_2_ (Sigma #C5670).
- 5 M NaCl (Sigma #S5886).
- 250 mM NiSO_4_ (Sigma #656895-10G).
- Imidazole (Sigma #I5513).
- HEPES (Sigma #H4034-500G).

### 3 Lipofectamine 3000 Transient Transfection of Gzms and GNLY

- Lipofectamine 3000 (ThermoFisher Scientific # L3000008).
- Opti-mem (ThermoFisher Scientific # 31985062).
- Coomassie Safe Blue stain (ThermoFisher Scientific # LC6060).
- Any kD™ Mini-PROTEAN^®^ TGX™ Precast Protein Gels, 12-well, 20 µl (Bio-Rad #4569035).
- Laemmli SDS sample buffer, non-reducing (4X) (Alfa Aesar #J63615-AC).
- 2-mercaptoethanol 14.2 M (Bio-Rad #1610710).
- Mini-PROTEAN Tetra Vertical Electrophoresis Cell for Mini Precast Gels, 4-gel (Bio-Rad #1658004).
- Precision Plus Protein™ Kaleidoscope™ Standards (Bio-Rad #1610375EDU).

### 4 Purification of Gzms From Culture Supernatant by Nickel-Immobilized Metal Affinity Chromatography (IMAC)

- Ni Sepharose beads (Cytiva # 17531806).
- Manually packed econo chromatography column 1.5 x 10 cm (Bio-Rad # #7371512).
- Suitable HPLC machine. Highly recommended to perform purification at 4°C to increase protein stability.

### 5 Enterokinase (EK) Treatment

- Centrifugal Filter Unit ≤10 kDa MWCO (Millipore UFC901008) for GzmA/B and ≤3 kDa MWCO (Millipore #UFC900324) for GNLY.
- Recombinant human EK expressed in CHO cells, suitable for cell culture and endotoxin tested (Sigma # SRP3032).
- Slide-A-Lyzer 10 kDa MWCO for GzmA/B (ThermoFisher Scientific #66455) or 3.5 kDa (ThermoFisher Scientific #66330) for GNLY.

### 6 Final Clean-up by MonoS Column (Cation Exchange Chromatography)

- MonoS-column (Cytiva #17516801).
- Endotrap column (Lionex #LET0009).
- 0.5 ml screw cap tubes (Midsci #PR-SC5AC2).

### 7 Characterization of Final Product

- Pierce™ Silver Stain Kit (ThermoFisher Scientific #24612).
- Trans-Blot^®^ Turbo™ Transfer System (Bio-Rad #1704150EDU).
- Trans-Blot^®^ Turbo™ Mini PVDF Transfer Packs (Bio-Rad #1704156EDU).
- For GzmA:
  - Human Granzyme A Antibody (R&D #MAB2905) at 1:250 in blocking buffer (5% milk in Tris Buffered Saline with Tween 20-TBST).
  - Z-L-Lys-SBzl hydrochloride (BLT substrate for GzmA) (Sigma #C3647-25MG).
  - 5,5′-Dithiobis (2-nitrobenzoic acid) (DTNB) (Sigma #D8130-500MG).
  - EasySep™ Human Monocyte Isolation Kit (Stemcell #19359).
  - RPMI 1640 Medium (ThermoFisher Scientific #11875093).
  - Human serum (Sigma #H4522-100ML).
  - Saponin from quillaja bark (Sigma #S7900).
  - BD Difco Dehydrated Culture medium: Middlebrook 7H9 Broth (BD #271310).
  - BD BBL Dehydrated Culture medium: Middlebrook ADC Enrichment (BD #211887).
  - Uridine, (5,6-^3^H) (PerkinElmer #NET367).
  - Illumina Gold F scintillation fluid (PerkinElmer #6013321).
  - MicroBeta^2^ Microplate Counters for Radiometric and Luminescence Detection with 1-detector (PerkinElmer #2450-0010).
  - Microbeta filtermat-96 cell harvester (PerkinElmer # D961962).
  - 8 x 12 Filtermat A, GF/C, 100/pk (PerkinElmer #1450-421).
- For GzmB:
  - BAADT Granzyme B substrate (AAD; Enzo Life Sciences #ALX-260-050-M005).
  - AAD assay buffer: H_2_O containing: 50 mM Tris-Cl, pH 7.5, 0.2 mM BAADT (AAD) from 20 mM stock solution in DMSO, 0.22 mM of 5,5’-dithio-bis (2-nitrobenzoic acid) (DTNB; Sigma #WXBD5644V) from 0.55 M stock solution in DMSO.
  - Anti-human Granzyme B Antibody (ThermoFisher scientific #14-8889-82) at 1:2000 in blocking buffer (3% BSA in TBST).
  - An5 buffer H_2_O containing: 10 mM HEPES pH 7.5, 140 mM NaCl, 2.5 mM CaCl_2_.
  - Buffer C Hanks’ balanced salt solution (HBSS) 10 mM HEPES pH 7.5, 4 mM CaCl_2_, 0.4% (w/v) bovine serum albumin (BSA).
  - Buffer P Hanks’ balanced salt solution (HBSS) 10 mM HEPES pH 7.5.
  - APC Annexin V Apoptosis Detection Kit with PI (BioLegend #B327051).
  - Perforin 10 μM stock for granzyme mediated apoptosis assay.
- For GNLY:
  - 6x-His tag antibody (HIS.H8) (Invitrogen #MA1-21315).

## Methods

Summary of the steps needed for purifying cytotoxic granular proteins:

### 1 Production of the Expression Plasmid pHLsec-Gzm

- Prepare total RNA from human NK cells using a suitable RNA isolation method and reverse transcribe using a first-strand cDNA synthesize kit. Amplify Gzm cDNA using PCR and clone into pHLsec (39) using the AgeI and KpnI sites.
- For GNLY shift from His tag at the N-terminus (but also applied to Gzms), the forward primer HisEk.consAge1For and the reverse primer GNLYSTOPkpn1Rev were used with the template DNA pHLsecGNLY. The transformation of DNA was done by the high efficiency transformation protocol NEB alpha with C2987I NEB 5­alpha competent *E. coli* cells. After overnight incubation the colonies were harvested. The obtained DNA was sequenced to confirm the cloning process.
- Confirm correct inserts by sequencing. Expand the expression plasmids in DH5α cells and purify using an endotoxin-free plasmid isolation kit and follow the manufacturer’s instructions.
- Resuspend the purified plasmids in endotoxin-free, sterile water at a concentration of 2 mg/ml and store at -80°C until use.

### 2 Expansion of HEK293T Cells

- Preparation of reagents:
  - Preparation of standard culture medium. To Dulbecco’s Modified Eagle Medium (DMEM) add 10% of standard fetal calf serum (FCS) and 1% penicillin-streptomycin. Filter with a 0.22 μm filter.
  - Prepare transfection medium, which is culture medium without penicillin-streptomycin.
  - Prepare the solutions described in **Table 1** and filter with a 0.22 μm filter. Receiver bottle should be single-use plastic. For water, use MilliQ water or other sterile and endotoxin-free equivalents to avoid endotoxin contaminations.
- Expanding HEK293T cells.
  - Grow HEK293T cells in 10 ml culture medium using 10 cm tissue culture dishes.
  - Split cells at 80% confluency (split-ratio of 1:4, usually every 3rd day). Cells loosely attach to dishes and can be mechanically detached without trypsinization by rigorously pipetting up and down. The following trypsinization method allows for a more accurate monitoring of cell heath during cell passaging and cell plating for the next step, so it is our preferred method for passaging cells. To do this, remove medium, wash once carefully with 5 ml of room temperature PBS, then add 2 ml of Trypsin 0.05% EDTA and incubate inside 37°C incubator for 2 min. Add 10 ml of complete medium to quench reaction, remove all cells from dish and collect in 50 ml conical tube. Spin down at 400 x g for 5 minutes at 22°C. Discard supernatant, count the cells and plate in 10 cm tissue culture dishes.
  - Plate cells the night before transfection to give 60-70% confluency at the day of transfection (seed around 5e^6^ cells per 10 cm plate in transfection medium-no antibiotics). A typical preparation size consists of 20 to 25 culture dishes with the expected yield around 700 μg of pure protein per plate.

**Table 1:** List of reagents necessary for the purification of Gzms and GNLY.

Stock solutions	
2 M Tris, pH 8
2 M CaCl_2_
5 M NaCl
250 mM NiSO_4_		
**Buffer solutions**		
Name		Components
His-binding buffer A		250 mM NaCl, 50 mM Tris, 10 mM Imidazole pH 8.0
His-binding buffer B		250 mM NaCl, 50 mM Tris, 1 M Imidazole, pH 8.0
EK Buffer		154 mM NaCl, 50 mM Tris, 4 mM CaCl_2_, pH 7.4
MonoS-binding buffer A		154 mM NaCl, 50 mM HEPES, pH 7.4
MonoS-binding buffer B		1 M NaCl, 50 mM HEPES, pH 7.4

### 3 Lipofectamine 3000 Transient Transfection of Gzms and Granulysin

- For transfection, ensure that cells are confluent around 70-90% as per L3000 product recommendations.
- Perform transfection using lipofectamine 3000 kit workflow. Use Opti-Mem for diluting L3000, DNA, and P3000. Do not mix L3000 with DNA directly because the DNA will precipitate, which will affect the transfection efficiency. First, dilute L3000 in one tube and in a separate tube dilute the DNA followed by addition of P3000. Use a ratio of 1:3 (DNA: lipofectamine). For a typical transfection, we suggest performing the transfection in 10 plates at a time to ensure that the timing between DNA-lipid complexing and addition to plates does not extend over 15 minutes. For more information, refer to manufacturer recommended settings.
  - For 10 plates, use 15 ml tubes:
    1. Tube 1: 810 μl L3000, 5 ml Opti-mem.
    2. Tube 2: 5 ml Opti-mem, 135 μl DNA at 2 mg/ml (270 μg total), mix, then 540 μl P3000.
    3. Mix tube 1 with tube 2 by inverting three time (no vortexing) and leave at room temperature for 10-15 minutes.
- After 10 minutes, aliquot 1.15 ml of lipid:DNA complexes to each plate. This should not exceed the total incubation time of 15 minutes per reaction tube.
- Analyze transfection efficiency by taking 20 μl of supernatant at day 3 (D3) and D4 post-transfection for SDS-PAGE and staining with Coomassie Safe Blue stain.
- Typically, produced protein can be harvested at D4 post-transfection. Cells can be replenished with fresh growing medium and harvested again at D8 [cells can produce secreted protein up to D10 (39)]. D4 harvest can be stored at -20°C and thawed at 4°C overnight at D7 so it is ready to be combined with D8 harvest.

### 4 Purification of Gzms From Culture Supernatant by Nickel-IMAC

- Decant the cell culture harvested supernatants into 250 ml tubes and clear by centrifugation. Spinning at 400 x g, 10 min, 4°C will clear the medium from detached cells. These detached cells can be re-added to original dish in fresh medium to continue to produce the desired protein (data not shown). Transfer the clarified supernatant into fresh 250 ml tubes and spin at 4,000 x g, 30 min at 4°C to remove any remaining cellular debris.
- Add 5 ml of 5 M NaCl, 6.25 ml of 2 M Trizma hydrochloride solution pH 8 and 1 ml of 0.25 M NiSO4 per 250 ml of cleared supernatant. Filter the supernatant using a 500 ml vacuum filter unit (0.45 or 0.22 μm).
- Equilibrate all 5 ml Ni Sepharose beads. First wash them with water, and then with His-binding buffer A. Add the beads to the filtered supernatant, with the magnetic stir bar to bind the His-tagged protein to the resin overnight at 4°C (batch-mode).
- Pellet the beads in 250 ml tubes and add to manually packed 1.5 x 10 cm column. Attach the column to a suitable FPLC system at 4°C.
- Wash the column with His-binding buffer A at a flow rate of 0.5 ml/min until UV absorbance (A_280_) baseline is reached (usually 10 Column Volumes or CV). At zero absorbance, start the elution process.
- Elute proteins with a linear 60 ml gradient 0-100% Buffer B (10 mM to 1 M imidazole) at a flow rate of 1 ml/min while collecting 2 ml fractions. Analyze the elution fractions by SDS-PAGE and Coomassie staining. Loosely bound proteins to Nickel-IMAC column will elute very early on, so once at 10% Buffer B, run can be put on hold (machine running at constant % Buffer B) so that weakly bound proteins (serum proteins) can be eluted. Once the chromatogram returns to baseline, then the rest of the linear gradient can be resumed.

### 5 EK Treatment

- Pool desired protein containing fractions in a spinning dialyzing tube with 10 kDa MWCO for Gzms or 3 kDa for GNLY. Typical centrifugation protocol consists of spinning the tube at 3,000 x g for at least 15 minutes at 4°C. Store a small sample at -20°C as pre-EK control.
- Add 5 μg of EK directly to the pooled fraction in the dialysis tube and dialyze overnight (at least 16 hr.) at Room Temperature (RT) in EK-buffer (3 L) using dialyzer (10 kDa for Gzms and 3 kDa for GNLY).
- After incubation, change the EK buffer with fresh buffer, take small sample of EK-treated protein, and add 5 μg more of EK.
- Analyze EK treated-protein and compare to the pre-EK control by SDS-PAGE and Coomassie staining. A protein band shift is evidence of N-terminal processing.
- When N-terminal processing is complete, change dialysis buffer to MonoS buffer A (3 L) and dialyze for another 1-2 hrs at 4°C. Filter dialysate (0.45 μm).

### 6 Final Clean-up by MonoS Column (Cation Exchange Chromatography)

- Equilibrate a MonoS-column with MonoS buffer A. Load the sample on the column with a flow rate of 0.5 ml/min at 4°C.
- After sample loading, wash the column with MonoS buffer A until UV absorbance baseline is reached (about 10 CV). Elute the proteins with a 30 ml linear gradient (150 to 1,000 mM NaCl). GzmA elutes at ~650 mM NaCl, GzmB at ~700 mM NaCl and GNLY at ~750 mM NaCl.
- Analyze elution fractions by SDS-PAGE and colorimetric assays (see below). Pool fractions containing proteins and concentrate (about 30-fold, to a concentration of at least 100 μM) in spin filters (15 ml, 10 kDa MWCO for Gzms and 3 kDa for GNLY).
- Optional:
  - Remove potential endotoxin presence (although attention should be taken throughout purification process not to introduce any) by adding concentrated sample into Endotrap column. Concentrated sample should be diluted at least 1:10 in Endotrap equilibration buffer (20 mM HEPES, 150 mM NaCl, 0.1 mM CaCl_2_, pH 7.5). Collect liquid after 1^st^ CV and concentrate in a sterilized spin filter (one rinse with 0.1N NaOH followed by wash with endotoxin free water and one wash with storage buffer). After concentration, protein sample can be diluted into desired storage buffer, and then concentrated again. Storage buffer for most of our applications is MonoS Buffer A (50mM HEPES, 154mM NaCl, pH 7.4) as we have not measured any buffer interference in our biological assays in terms of cytotoxicity and interaction with primary cells. Our proteins are concentrated to around ~700 μM, and for most of our applications the concentration needed is between 20 pM and 200 nM (1:3,500-35,000,000 dilution into final reagent).
- Aliquot the concentrated Gzm preparations and store at -80°C in screw cap tubes to avoid sample loss.

### 7 Characterization of Final Product

- For all purified cytotoxic granule components:
  - SDS-PAGE followed by both Coomassie and Silver staining to detect potential protein contaminants, as well as Western Blot analyses to confirm identity of detected proteins.
  - Colorimetric assays to measure protein substrate cleavage efficiency (or to monitor loss of enzymatic activity in protein variants).
  - Biological assays measuring known biological effects mediated by purified proteins.
- For GzmA:
  - SDS-PAGE (silver staining and western blot) both under reducing and non-reducing conditions to confirm homodimer presence and correct protein folding.
  - BLT esterase assay-protocol and calculation of specific activity adapted from (17):
    1. Substrate Z-L-Lys-SBzl hydrochloride is added to 96 well plates with a serial dilution between 19.5-2,500 µM. Assay buffer is 50 mM Tris, 154 mM NaCl, pH 7.5 in presence of 0.55 M (5,5-dithio-bis-(2-nitrobenzoic acid) (DTNB) chromophore. 120 pM of protein is added per well and substrate hydrolysis is quantified by measuring the absorbance at 405 nm using plate reader. Esterolytic activity is reported as rate of hydrolysis using extinction coefficient of 13,100 M^-1^cm^-1^ for the 3-carboxy-4-nitrophenoxide ion. Specific activity is measured as nM product/min/nM of enzyme present.
  - Mycobacterial Growth Inhibition Assay-protocol adapted from (17):
    1. Primary CD14^+^ monocytes are thawed from Peripheral Blood Mononuclear Cells (PBMC) and plated in round-bottom 96-well plates in R+2 (RPMI-1640 + 10% human HAB serum + 1% l-glutamine). The monocytes are then infected with Connaught BCG (Multiplicity of Infection = 3) and treated with 200 nM GzmA. After overnight infection, cells are gently washed with R+2 medium three times to remove extracellular BCG and resuspended in R+2 medium. After 72 h co-culture, cells are lysed with saponin solution in RPMI-1640, and the reaction is quenched after 2 h with 100 µL 7H9+ADC containing 1 µCi 5,6-^3^H-uridine. After 72 h, plates are harvested onto glass fiber filter papers (filtermats). Filtermats receive Illumina Gold F scintillation fluid and are imaged using a MicroBeta^2^ liquid scintillation counter that measures Disintegration Per Minute (DPM). The % inhibition is calculated as: 100 – 100 x (DPM from wells treated with GzmA and infected with BCG/DPM from wells infected with BCG).
- For GzmB:
  - SDS-PAGE (silver staining and western blot).
  - AAD assay: 5 μl of FPLC fractions or 400, 100, 25 nM concentrations of recombinant GzmB with comparison to native samples are combined with 200 μl of AAD assay buffer in a 96-well flat-bottom plate. Followed by incubation at 37°C for 5 minutes, the spectrophotometric cleavage activity of the active protein is measured by absorbance at the optical density of 405 nm.
  - Granzyme mediated Annexin-V/Propidium iodide (PI) assay: The target cells (Jurkat) are washed once with 5 ml buffer C and resuspend at 10^5^ cells/well in a 96-well V-bottom plates in 30 μl buffer C. Perforin and purified recombinant GzmB is diluted in 30 μl buffer P to 2X the sublytic dose and added to the cells along with PFN only, granzyme only, and buffer-only treated cells. The cells are incubated for 60 min at 37°C and 100 μl of An5 buffer is added to each well. After centrifugation for 3 min at 500 x g, the cells are resuspended in 100 μl An5 buffer containing APC-conjugated Annexin V (1:33 dilution) and incubated for 10 min at room temperature in the dark. The cells are washed once in 100 μl An5 buffer and resuspend in An5 buffer containing 2 μg/ml propidium iodide and analyzed by flow cytometry (35).
  - Biological assay is the ability of GzmB in combination with GNLY to suppress the growth of extracellular *E. coli*. 250 nM of GzmB are incubated with 100 nM of GNLY and bacterial growth is measured over time by OD_600_ readouts (26, 27).
- For Granulysin:
  - Western Blot that looks at His-tag presence using Anti-His antibody. Thus, EK cleavage efficiency can be monitored as final product will become Anti-His negative.
  - Biological assay is the ability of GNLY to suppress the growth of extracellular *E. coli*. 100 nM of GNLY is incubated and bacterial growth is measured over time by OD_600_ readouts (26, 27).

### Statistical Analysis

For generation of graphs and statistical analysis we used GraphPad Prism version 9.0.0 for Mac, GraphPad Software, San Diego, California USA, www.graphpad.com.

## Results

### Improved Purification Process for the Production of Gzms

**Figure 1:**
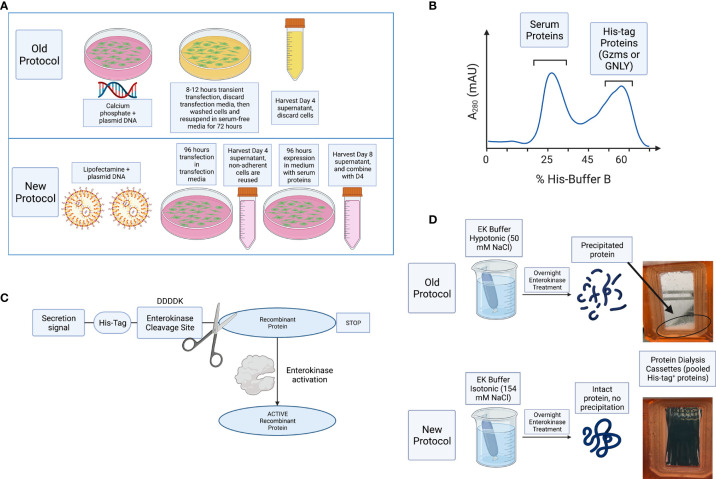
Schematic representation of improvements and summary of steps necessary for purification of Gzms and GNLY. **(A)** Comparison of the old vs new protocols, HEK293T cells are transiently transfected with lipofectamine 3000 instead of calcium phosphate. Cells are then incubated for 96 hours in transfection medium without the need for washing away the calcium phosphate transfection solution with PBS, and subsequent resuspension in serum-free medium. Secreted protein is harvested at D4, cells are resuspended in fresh medium, and harvested again at D8. **(B)** Secreted proteins are then purified using Ni-IMAC purification by isolation of His-tag on recombinant proteins and removal of bulk serum proteins. **(C)** Scheme showing that following EK activation, the His-tag is removed from the final recombinant protein. **(D)** EK cleavage under isotonic solution activates final protein and prevents sample loss, while hypotonic solution leads to protein precipitation and loss of sample recovery. Created with BioRender.com.

We have modified several steps in the recombinant protein expression and purification protocol for GzmA and GzmB (38), with the process schematically represented in **Figure 1**. As shown in **Figure 1A**, the switch from calcium phosphate to lipofectamine delivery allowed us to use significantly less cells (5e^8^ cells vs 1.75e^8^ cells), and correspondingly less plasmid DNA (2 vs 0.27 mg). In parallel, the previous transient transfection procedure included only 8-12 hours incubation before changing to a serum-free medium, while our new transfection protocol extends the incubation in medium with serum for 96 hours before adding fresh medium on D4. The previous method included the use of Ex-cell HEK293 serum-free medium (Sigma # 14571C-500ML), which allowed transfected cells to produce protein and proliferate (data not shown).

**Figure 2:**
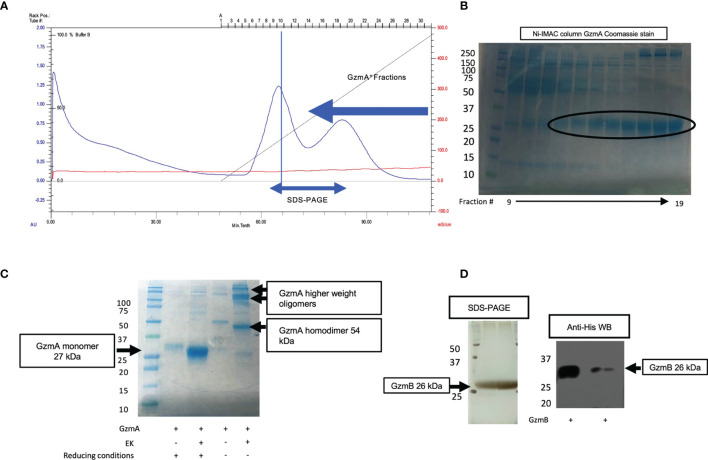
Ni-IMAC purification and EK activation of Gzms. **(A)** Representative chromatogram of a Ni-IMAC column run for GzmA (similar layout for GzmB) showing elution of serum proteins first (starting at fraction 6, peaking at 10), then elution of GzmA^+^ fractions (starting at fraction 13 and ending at fraction 28). **(B)** Coomassie stained SDS-PAGE under reducing conditions (GzmA monomer pre-EK activation runs at 31 kDa). On the left, Molecular Weight (MW) markers. **(C)** Example of band shift for GzmA following EK activation after overnight incubation. GzmA monomer on the left (under reducing conditions) transitions from 31 kDa to 27 kDa, while GzmA homodimer on the right transitions from 62 kDa to 54 kDa. Other bands other than GzmA monomer and homodimer reflect oligomers of GzmA (multiples of GzmA homodimer and of unknown significance). On the left, MW markers. **(D)** On the left, loading control of pre and post-EK GzmB by silver staining, and on the right the final product displays a profoundly reduced His-tag as shown for GzmB (26 kDa) by Anti-His WB. The significantly lower band intensity on the right is indicative of near complete EK cleavage. On the left, MW markers.

For improved purification, D4 and D8 supernatants were combined, and cell debris removed by centrifugation and filtration. The combined supernatants were incubated overnight with nickel Sepharose beads and then washed with His-buffer A to remove weakly bound proteins. As shown schematically in **Figure 1B** and in a representative image of A_280_ monitoring during His-buffer B elution in **Figure 2A**, the bound proteins are eluted with a linear gradient from 0-100% of His-buffer B, corresponding to 10-1000 mM imidazole. When 40% His-buffer B (~400 mM Imidazole) is achieved, Gzms start to elute. SDS-PAGE followed by Coomassie stain is used to identify which fractions to combine for the next steps as shown in **Figure 2B**.

Pooled and concentrated fractions are then incubated overnight with EK to activate the proteins as represented schematically in **Figure 1C**. It was originally noted that EK cleaves more efficiently at low salt concentrations (e.g., 50 mM NaCl) (38, 40). However, we observed significant protein precipitation after overnight dialysis in hypotonic solution as shown in **Figure 1D**. Thus, we altered the EK buffer to an isotonic solution which prevented protein precipitation and allowed EK to cleave Gzms. The recombinant human EK from CHO cells used in our protocol can cleave and activate target proteins in isotonic buffer conditions as shown by the band shift of Gzms in **Figure 2C** and the loss of the His signal by western blot in **Figure 2D**. On the left of **Figure 2D**, silver stains show that samples pre and post-EK were comparable in terms of protein loading, while on the right, the EK-cleaved GzmB sample shows that only a significantly small portion of total prep remains uncleaved. The recombinant Gzm plasmids were engineered to express the 6x poly His Tag at the N­terminus of the DNA upstream of the EK cleavage site. This is an optional step that allows the researcher to monitor EK activation efficiency by probing post-EK protein with Anti-His antibody; a lower molecular weight confirms that the secretion signal containing the His tag has been cleaved. This shift in MW is more evident with homodimeric GzmA as two His tags are cleaved, while monomeric GzmB has only one.

**Figure 3:**
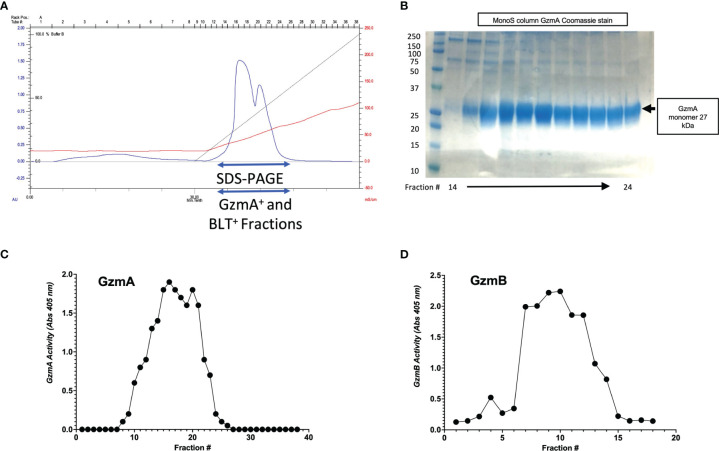
MonoS purification of Gzms. **(A)** Representative chromatogram of MonoS run for GzmA (similar layout for GzmB) showing elution of GzmA around 650 mM NaCl (e.g., fractions 12 through 27). **(B)** Fractions 14 through 24 were probed by SDS-PAGE under reducing conditions followed by Coomassie staining, which show GzmA monomer at 27 kDa. On the left, MW markers. **(C)** Substrate activity assay for GzmA (BLT assay) using the MonoS fractions, where substrate is in excess compared to protein. **(D)** Substrate activity assay for GzmB (AAD) using MonoS fractions.

**Figure 4:**
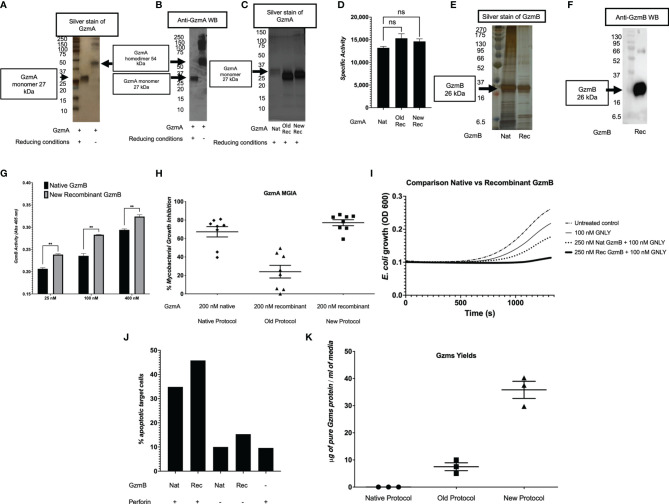
Purity and yield of Gzms. GzmA purity was assessed by silver stain **(A)** and Western Blot (WB) **(B)** under reducing conditions showing GzmA monomer at 27 kDa, and non-reducing conditions showing GzmA homodimer at 54 kDa. Under non-reducing conditions, GzmA homodimer and multimers are present. On the left, MW markers. **(C)** Silver stain comparing Native GzmA [purified per (35)], Recombinant GzmA purified using old protocol vs new protocol under reducing conditions. Monomer band shows at around 27 kDa and on the left MW markers. **(D)** Specific activity of GzmA after analyzing the enzyme kinetics at cleaving substrate BLT and comparison between native, old protocol rec GzmA and new protocol rec GzmA (data representative of three independent experiments; ns calculated using student t test; means and SD). **(E, F)** present similar purity results for GzmB and comparisons between native (purified per [35)] and new recombinant purification (GzmB runs at 27 kDa). On the left, MW markers. **(G)** Comparison of native vs new recombinant GzmB’s enzymatic activity as measured by cleavage of AAD at different protein concentrations (n=3 independent experiments, mean and SD, student t test). **(H)** GzmA-mediated functional potency as measured by the MGIA, and comparison between proteins purified with the native method, the old recombinant method, and the new recombinant method (n=8 human subjects, data representative of at least two independent experiments; means and SEM). **(I)** GzmB suppresses extracellular *E.coli* growth after delivery into bacteria with added GNLY (averaged data representative of at least two independent experiments). **(J)** Background adjusted percentage of apoptotic cells as measured by Annexin V and PI staining after incubation of proteins with Jurkat cells (n=2, mean). **(K)** Comparison of yields of Gzms (GzmA and GzmB) between native, old, and new protocols (data representative of multiple independent purifications involving different operators and sites; means and SEM).

After confirmation of EK cleavage, proteins were dialyzed into MonoS-buffer A, and then separated as shown in the representative chromatogram shown in **Figure 3A**. As shown in **Figure 3B**, the fractions contain predominantly the reduced GzmA monomers (~31 kDa) and were enzymatically active as measured by specific Gzm peptide substrates. The BLT assay is used for GzmA as shown in **Figure 3C** and for GzmB the AAD assay is used as shown in **Figure 3D**. Proteins are then concentrated, undergo endotoxin removal using Endotrap columns. As shown by silver stain and western blot in **Figures 4A, B**, GzmA is highly pure and able to form homodimers (as well as multimers – previously reported in (14, 17) and of unknown significance). For **Figure 4C**, the differences between native, recombinant GzmA purified using old protocol vs new protocol are analyzed for purity by silver stain. To confirm that all purified proteins are capable of cleaving substrates, the specific activity of each purified GzmA was compared and showed no difference at cleaving BLT as shown in **Figure 4D**. In **Figures 4E, F**, highly purified GzmB is shown by silver stain and western blot. Like GzmA, in **Figure 4G** we compared Native to New recombinant GzmB enzymatic activity at different protein concentrations, and it appears that recombinant GzmB performs better than native counterpart, probably owing to a purer final product. In **Figure 4H**, the biological activity of GzmA in a Mycobacterial Growth Inhibition Assay (MGIA). While the old purification protocol produced inhibitory GzmA [purified per (38)], the new protocol yields more biologically potent GzmA similar to native GzmA [purified per (35)]. Similarly, in **Figure 4I**, recombinant GzmB produced using this new protocol performed significantly better than native GzmB [purified per (35)] at suppressing extracellular bacterial growth after delivery into the organisms with GNLY addition. To further compare native vs new recombinant GzmB, we performed an Annexin V and PI staining of target cells incubated with and without perforin, and the results are shown in **Figure 4J**.

In **Table 2**, we compared the cell expansion times, total purification times, purity levels, advanced equipment necessary, yields per purification, DNA amounts needed, costs per purification, and the overall efficiency. As shown, our updated protocol provides many advantages compared to other protocols (35, 38). As shown in **Figure 4K**, our system has allowed us to increase the final yields of purified Gzms compared to the yields obtained with other protocols (35, 38).

**Table 2:** Comparisons between native, old recombinant (38), and new recombinant protocols.

	Native Protocol		Old Recombinant Protocol (Dotiwala, 2015)		New Recombinant Protocol	
Expansion of Cells Time	3 weeks (NK92MI cells)		10 days		7 days	
Purification Time	3 days		3 days		3 days	
Advanced Equipment Necessary	Bioreactor; Cavitation Bomb with Nitrogen Tank		–		–	
Yield per Purification	~50 μg from 2e^9^ NK92MI cells		~1.5 mg from 5e^8^ HEK293T cells		~15 mg from 1.75e^8^ HEK293T cells	
Plasmid DNA Needed	N/A		2 mg		0.27 mg	
Cost per Purification in USD as of 2022	3 weeks NK92MI growing medium	$312	10 days HEK293T growing medium	$45	7 days HEK293T growing medium	$45
			Calcium phosphate transfection reagents (reagents + DNA)	$50 for reagents + $200 for DNA	Lipofectamine transfection cost (reagent + DNA)	$450 for reagents + $50 for DNA
			1L serum-free media	$111		
Comparative Total Costs ($/ug)	~6.24		~0.27		~0.04	

### GNLY Purified With the Mammalian Expression System Is Biologically Active

**Figure 5:**
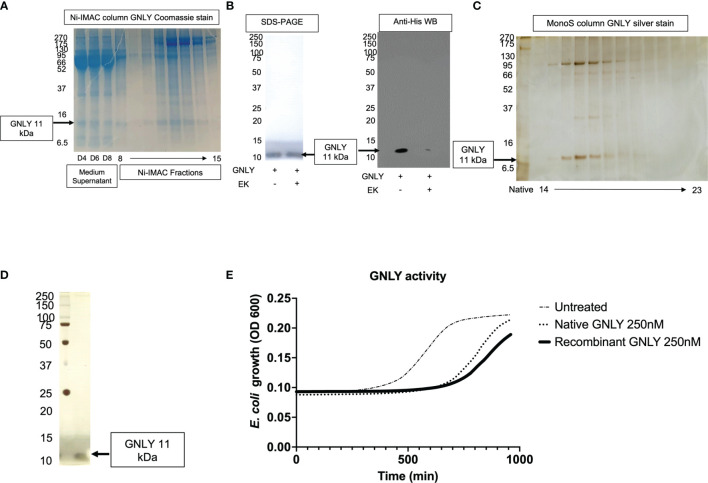
Mammalian GNLY production: purification, activation, and characterization. **(A)** GNLY (11 kDa bands) is secreted into the supernatants of transiently transfected cells and detected at D4, D6, and D8. Ni-IMAC fractions are tested by Coomassie-stained SDS-PAGE to confirm GNLY separation. On the left, MW markers. **(B)** Loading control for GNLY on the left under SDS-PAGE, while on the right GNLY activation EK activation (loss of His-tag). On the left, MW markers. **(C)** Native GNLY in lane 2 for comparison and MonoS fractions (14 through 23) after silver staining. On the left, MW markers. **(D)** GNLY purity was assessed by SDS-PAGE after GNLY^+^ MonoS fractions were pooled together. On the left, MW markers. **(E)** GNLY-mediated inhibition of *E*. *coli* and comparison between native and recombinant samples using 250 nM of GNLY.

GNLY was purified using the improved protocol as summarized in **Figure 1** and production was monitored in **Figure 5** using secreted protein supernatants. Similar to Gzms, GNLY elutes from Ni-IMAC column around 40% His-buffer B. In **Figure 5A**, the medium supernatant at D4, D6, and D8, and the Ni-IMAC fractions show GNLY presence after SDS-PAGE and Coomassie staining. The fractions containing GNLY were pooled for subsequent EK treatment and further purification. In **Figure 5B**, EK treatment cleaves the His-tag similarly as in **Figure 2D**. In **Figure 5C**, the GNLY MonoS fractions are shown as well as the native GNLY protein [purified per (35)] in the second lane for comparison. Finally, in **Figure 5D**, pure GNLY is shown by SDS-PAGE and silver stain.

The final yield obtained was 7 mg from 10 - 10 cm^2^ plates of HEK293T cells (from 200 ml of total supernatant giving a final yield of 35 μg pure protein per ml of supernatant, which is in line with the Gzms purifications). Pooled GNLY fractions are then tested in the antimicrobial assay (26, 27). *E. coli* were treated with 250 nM of native vs recombinant GNLY or left untreated before monitoring bacteria viability in growth assays. Recombinantly expressed GNLY appears to better inhibit bacterial growth as shown in **Figure 5E**.

## Discussion

The purification in high yields of cytotoxic granular proteins in a reliable and consistent manner has been a significant barrier in the field of immunology. Early native purification protocols did not exclude contamination with other granular proteins in the final products (32). For instance, GzmA was routinely co-purified with Granzyme K, and could not be distinguished as they both cleave the BLT substrate. Only with the commercialization of high-quality monoclonal antibodies against GzmA and GzmK have researchers been able to separate fractions to avoid potential co-contaminants from native purifications (35). The sequencing of the human genome and the development of cDNAs that include all human Gzms and GNLY allowed the recombinant expression of these proteins first in bacteria (29–31), later in yeast (41, 42), and finally in insect cells (33, 43). While all these methods allow protein production, bacterial expression is associated with contamination of the final product with endotoxins (44), skewing assay results particularly when working with cells that are sensitive to this stimulus. *Pichia pastoris* expression does not produce endotoxin, however, like bacterial expression systems, the granzyme products do not normally have native glycosylations known to be important for normal protein physiology (45). While monitoring and comparing post-translation modifications in Gzms between native, old recombinant, and new recombinant protocols was beyond the scope of this work, it is important to point out that there continues to be a knowledge gap on their role in biology as highlighted in (4, 46, 47).

In this work, we have used transient transfection in a mammalian expression system using HEK293T cells, lipofectamine 3000, and isotonic buffers. Compared to previous work (38), our protein products (GzmA, GzmB and GNLY) are purified in large yields, with a cost-efficient method, are more biologically active, and are therefore useful in complex immunological assays. Previous expression of GzmB through stable transfection gave purification yields of ~4 mg/L of culture supernatant (45), while our new method yields much larger amounts up to 40 mg/L of culture supernatant. Our process involves a faster purification, results in high purity products, does not involve the use of advanced equipment, provides larger yields, needs less plasmid DNA, and overall is significantly more cost-effective (**Table 2**). The lower amount of DNA allowed us to use Maxi instead of Giga DNA preps to purify plasmid DNA, which further decreased our protein expression cost. The main improvements in our protocol are: 1) the transition from calcium phosphate transfection to lipofectamine 3000, 2) the use of transfection medium that contains serum proteins for optimal cell viability, 3) the extended production over 96 hours, 4) the use of isotonic buffers throughout the purification process, and 5) the translocation of the His tag upstream of EK site for monitoring protein activation (**Figure 1**). Use of lipofectamine 3000 or similar reagents are highly recommended for higher transfection efficiencies than lipofectamine 2000 (48). These changes have significantly improved the yield of our protein purifications as shown in **Figure 4G** and allowed for the purification of recombinant human GNLY (**Figure 5**). Our recombinant system also facilitates site-directed mutagenesis and has been successfully employed to mutate a key amino acid within the active site of Granzyme A (GzmA-S195A) (17).

There were previous reports of GNLY expression in bacteria (1, 49), yeast (32), and insect cells (33), but to the best of our knowledge, this is the first time that GNLY has been successfully purified in a mammalian expression system. As shown in **Figure 5E**, GNLY expressed with our method has greater antimicrobial activity compared to previous purifications. Similarly, as shown in **Figures 4E, F**, GzmA and GzmB purified with this updated protocol give a better biological response at inhibiting intracellular mycobacterial growth (GzmA) and suppressing extracellular bacterial growth (GzmB) than the previous purification protocol.

The construct that was used in our purification process uses an EK cleavage site at the N-terminus of the proteins. The overnight EK cleavage in hypotonic solution was believed to be required for optimal enzymatic activity (38). However, as shown in **Figure 1D**, most of the protein produced precipitates in this solution, suggesting that, at least for Granzyme A, salt concentration is key for proper protein folding. Previous studies hinted at this possibility (34), in which GzmA activity dropped by 66% when treated with hypotonic detergent washes. For GzmB, the activity drop was even greater (by 94%). Given the presence of granzymes in serum, we hypothesized that isotonic conditions would favor promotion of native protein structure. When comparing the old protocol to this new protocol, the role of hypotonicity in lowering activity is shown by decreased inhibition of intracellular mycobacteria as presented in **Figure 4E**.

Previous failed attempts at purifying active GNLY from a mammalian expression system could also have been due to the hypotonic conditions, and studies have also shown GNLY’s antimicrobial activity is influenced by salt concentration (25). Thus, we recommend maintaining human serum physiological conditions during the purification process (154 mM NaCl and pH 7.4) and storing the final product in 50 mM HEPES buffer. Our proteins are concentrated to ~700 μM and for most of our applications, the concentration needed is between 20 pM and 200 nM (1:3,500-35,000,000 dilution into final reagent).

The increase in biological activity for Gzms and GNLY (**Figures 4E, F**, **5E**) could be due to an increased purity of the final proteins, as well as the increased protein stability by use of this updated protocol. In fact, it is known that high protein concentration allows protein to remain more stable and in solution (50).

In conclusion, we have established an improved cytotoxic granular protein production process using a mammalian expression system that allowed us to purify large yields of Gzms and express human GNLY for the first time. This robust purification system allows the researcher to obtain enough protein material for in-depth studies to unravel unknown mechanisms involved in protection against infections and cancers, while also opening new doors for therapeutic applications. GNLY is considered as a potential alternative to antibiotics (51–53), while GzmB was found to be highly effective in limiting human tumor progression (28). The use of readily available equipment and reagents will allow researchers of all backgrounds to use these tools and significantly contribute to a better understanding of the function of these proteins and their translation to human medicine.

## Data Availability Statement

The raw data supporting the conclusions of this article will be made available by the authors, without undue reservation.

## Author Contributions

VR and OH designed and conducted experiments under the supervision of DH, MW, and SS. SB, DG, and PM provided logistical and technical support for the experiments represented in this article. VR, OH, DG, DH, and MW wrote and finalized this manuscript. All authors contributed to the article and approved the submitted version.

## Funding

Research reported in this publication was supported by the National Heart, Lung, And Blood Institute under Award Number F30HL151136 to VR, National Institute of Allergy and Infectious Diseases of the National Institutes of Health under Award Number R01AI048391 to DH, and the Swiss National Science Foundation (SNSF grant # 310030_169928), the Novartis Foundation for Medical-Biological Research and the Vontobel-Foundation to MW.

## Author Disclaimer

The content is solely the responsibility of the authors and does not necessarily represent the official views of the National Institutes of Health.

## Conflict of Interest

The authors declare that the research was conducted in the absence of any commercial or financial relationships that could be construed as a potential conflict of interest.

## Publisher’s Note

All claims expressed in this article are solely those of the authors and do not necessarily represent those of their affiliated organizations, or those of the publisher, the editors and the reviewers. Any product that may be evaluated in this article, or claim that may be made by its manufacturer, is not guaranteed or endorsed by the publisher.
